#  Transient Global Amnesia after Ablation of Premature Ventricular Beats Arising from the Right Coronary Cusp

**Published:** 2010-08-15

**Authors:** Rasoul Mokabberi, Chafik Assal, Haftbaradaran M Afsaneh, Randle Storm, Gopi Dandamudi

**Affiliations:** Geisinger Medical Center, Danville, PA

**Keywords:** Transient Global Amnesia, Ablation of Premature Ventricular Beats

## Abstract

A 58-year-old female underwent PVC ablation within the right coronary cusp for symptomatic PVCs and suspected PVC-induced cardiomyopathy. Immediately after the procedure, she started to complain about feelings of impending doom, disorientation to time and place, and amnesia regarding the procedure. No sensory or motor deficits could be elicited. A thromboembolic event was suspected and she was evaluated by a neurologist. CT scan of her brain was negative. She was diagnosed with transient global amnesia and her mentation returned to baseline within 4 hours after the procedure.

## Introduction

Radio frequency catheter ablation (RFCA) has become a major therapeutic option for symptomatic ventricular arrhythmias. Serious complications are generally rare, including pericardial effusion/tamponade, heart block, coronary artery injury, aortic valve damage, stroke, transient ischemic attack, vascular injury, and death [1]. The thromboembolic rate of a general electrophysiology study is reported to be 0.7-0.8%. The rate of thromboembolic events increases to 2.8% with ablation of ventricular arrhythmias [1]. We report a benign complication during RFCA of premature ventricular beats (PVCs) arising from the right coronary cusp. Transient global amnesia (TGA) has a dramatic presentation and may mimic an acute cerebral ischemic /embolic event.

## Case Report

**Figure 1:**
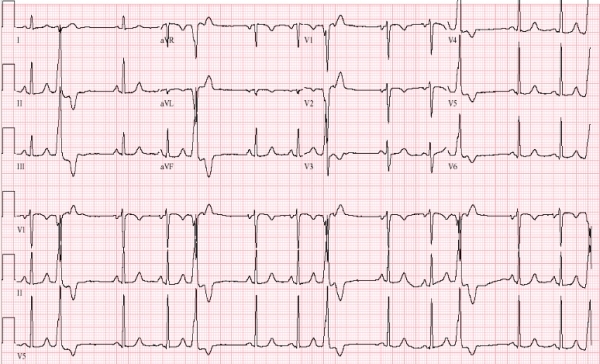
12-lead ECG demonstrating monomorphic PVCs with a left bundle branch block inferior axis pattern

A 58-year old female presented with a history of palpitations for 3 years.  She was starting to experience near syncopal episodes. Her physical exam was unremarkable. An exercise stress echocardiography showed an ejection fraction of 40-45%, frequent PVCs, but no evidence of ischemia. A 24-hour Holter monitor demonstrated approximately 36,000 monomorphic PVCs. Her 12 lead ECG showed monomorphic PVCs with a left bundle branch block, inferior axis pattern (Figure 1).  She was diagnosed with symptomatic PVCs and likely PVC-induced cardiomyopathy. After discussing her options including medical therapy versus RFCA, she decided to undergo ablation of her PVCs.

During the procedure, a right ventricular quadripolar catheter was used for pacing and surface ECGs were used as reference for mapping the PVCs. An AcuNav intracardiac ultrasound catheter (Biosense Webster, Diamond Bar, CA) was placed in the RV for visualizing the left ventricular outflow tract (LVOT) and the aortic cusps to assist with ablation.  A 4 mm D-curve NaviStar ablation catheter (Biosense Webster, Diamond Bar, CA) was used for mapping the RV and RVOT. 3-dimensisonal electroanatomic mapping using the CARTO system (Biosense Webster, Diamond Bar, CA) was used to perform an activation map of the RVOT. This demonstrated a diffuse area within the posteroseptal aspect of the RVOT that preceded the QRS onset by approximately 5 to 10 msec. However, pace mapping in these areas did not result in any good pace matches with the culprit PVC. Therefore, the ablation catheter was advanced into the LVOT via a right femoral arterial approach for mapping. Heparin bolus followed by an infusion was initiated to maintain an activated clotting time (ACT) around 250 seconds for the entire procedure. Earliest activation sites were mapped to the right coronary cusp and had the following characteristics; 1) local activation times preceded the surface QRS onset during PVCs by 20-22 msec; 2) Pace mapping in these areas demonstrated at least 11 out of 12 pace match with the culprit PVC. There were also fractionated electrograms with a good QS morphology within the unipolar electrogram. ICE images demonstrated that the ablation catheter tip was moving away form the right coronary ostium and an aortic root angiogram confirmed that these sites were at least 1 cm away from the right coronary ostium. Total duration of radiofrequency energy (RF) applications in the right coronary cusp was approximately 6 minutes, with a maximum power and temperature of 30 Watts and 55 degrees Centigrade, respectively. With each RF application, patient would go into ventricular bigeminy followed by complete loss of PVCs. This would be maintained for a few minutes after cessation of RF, followed by recurrence. It was felt that continued ablation in this area would increase procedural risk and therefore, the procedure was concluded.  Patient was awake during the entire procedure.  Minimal intravenous sedation was used for the entire procedure (2 mg of midazolam and 40 mg of methohexital sodium). 

Her vital signs were stable at the end of the procedure. She was awake and alert but started to perseverate and kept asking multiple orienting questions. She also started to panic and complained about feelings of impending doom.  She was oriented to person but not to place and time. She was able to recall all of the events leading to entry into the hospital, but was amnestic surrounding the procedure itself. She continually perseverated for the next 2 hours, repeating the same questions and also complained of a headache. Her clinical exam in the electrophysiology lab did not demonstrate any gross focal weakness, numbness or lateralizing sensory changes. She  followed multiple-step commands without problem. There was no deficit in speech, language, or other cranial nerve function. Serum electrolytes, blood glucose level, CBC and CT scan of the brain (without contrast) were immediately done which were normal. Neurology consult was requested and she was diagnosed with transient global amnesia (TGA). Patient returned to her baseline mental status within 4 hours and was discharged home the following day. She did not have any neurological deficits at the time of discharge (except for a headache that resolved within 24 hours).

## Discussion

Like any invasive procedure, radiofrequency ablation carries some risk. However, the risk of complications is small in most cases.  We report a case of TGA as a benign complication of RFCA procedure for the first time. TGA is a sudden, temporary episode of memory loss that cannot be attributed to a more common neurological condition such as stroke or epilepsy. TGA is one of the most striking syndromes in clinical neurology whose key defining characteristic is temporary but causes almost total disruption of short-term memory with a range of problems accessing older memories [2]. A person in a state of TGA exhibits no other signs of impaired cognitive functioning but recalls only the last few moments of consciousness plus deeply-encoded facts of the individual's past, such as his or her own name. The onset of TGA is generally fairly rapid, and its duration varies but generally lasts between 2 to 8 hours [3]. A person experiencing TGA typically has memory only of the past few minutes or less, and cannot retain new information beyond that period of time. One of its bizarre features is perseverance in which the victim of an attack faithfully and methodically repeats statements or questions, complete with profoundly identical intonation and gestures "as if a fragment of a sound track is being repeatedly rerun" [4]. This is found in almost all TGA attacks and is sometimes considered a defining characteristic of the condition [3,5]. They usually tend to remain oriented to person but not to place or time. Our patient exhibited all of the above mentioned features post-procedure with complete resolution within a few hours.

TGA appears to be harmless and future recurrence is unlikey. Episodes are short-lived (less than 24 hours) and memory completely recovers. Additional signs and symptoms that may accompany TGA include headache, nausea, vomiting, dizziness, chills or flushing, fear of dying, pins-and-needle sensation, powerful expression of emotion, sweating, and chest or neck pain [6]. In a significant number of patients with TGA, a stressful precipitating factor can be identified [7]. Because the presentation of TGA can be dramatic and may mimic an acute cerebral ischemic event, a thorough neurologic evaluation should be pursued.  Risk factors for the development of TGA include age greater than 50 years and a history of migraines. The exact cause of TGA is unknown at this time, although an epileptic event, interruption to cerebral blood flow or migraines have been postulated. In this case, minimal sedation was used and may not be the likley cause of her symptoms.

## Conclusion

TGA appears to be a rare complication during electrophysiology procedures.  It can mimic an acute storke.  However, it also appears to have certain distinct characteristics such as perserveration and short term memory loss that can help distinguish it from acute ischemic/embolic cerebral events.  It also appears to carry a benign prognosis in the long-term.

## References

[R1] Wilber D, Natale A (2009). Ventricular Tachycardia/Fibrillation Ablation.

[R2] Sander K (2005). New insights into transient global amnesia: recent imaging and clinical findings. Lancet Neurol.

[R3] Hodges JR (1990). Syndromes of transient amnesia: towards a classification. A study of 153 cases. J Neurol Neurosurg Psychiatry.

[R4] Frederiks JA (1993). Transient global amnesia. Clin Neurol Neurosurg.

[R5] Butler CR (2008). Recent insights into the impairment of memory in epilepsy: transient epileptic amnesia, accelerated long-term forgetting and remote memory impairment. Brain.

[R6] http://www.mayoclinic.com/health/transient-global-amnesia/DS01022.

[R7] Bortolon RJ (2005). Transient global amnesia after general anesthesia. Anesth Analg.

